# Noninferiority Randomized Controlled Clinical Trial Assessing the Antiplaque Efficacy of Fatty Acids–Based Mouthrinse

**DOI:** 10.1002/cre2.70171

**Published:** 2025-07-09

**Authors:** Serena Altamura, Eleonora Ortu, Antonella Barone, Annalisa Monaco, Sara Di Nicolantonio, Martina Cardisciani, Davide Pietropaoli

**Affiliations:** ^1^ Department of Clinical Medicine, Public Health, Life and Environmental Sciences University of L'Aquila L'Aquila Italy; ^2^ Center of Oral Diseases Prevention and Translational Research Dental Clinic L'Aquila Italy; ^3^ Oral Diseases and Systemic Interactions Study Group (ODISSY Group) L'Aquila Italy; ^4^ Department of Medicine, Case Western Reserve University School of Medicine Cleveland USA

**Keywords:** dental caries, dental plaque, fatty acids, oral hygiene, periodontitis, prevention mouthrinse

## Abstract

**Objectives:**

The market offers many plaque‐controlling mouthrinse options, but recent research reveals fatty acids' antimicrobial potential. Despite limited evidence on their antiplaque effects, fatty acids are intriguing for oral care innovation.

**Material and Methods:**

This noninferiority randomized clinical trial assessed the antiplaque efficacy of a fatty acids–based (FAG) compared to stannous fluoride (SF) mouthrinse in experimental gingivitis induced by 14 days of oral hygiene cessation. Participants used assigned treatments twice daily for 14 days. Full Mouth Plaque and Bleeding Scores (FMPS/FMBS) served as primary outcomes. Statistical analyses encompassed parametric and nonparametric methods, as well as logistic regression models.

**Results:**

Thirty‐one volunteers (22.9 ± 1.6 years, 58.1 female) completed the trial, split between FAG (*n* = 15) and SF (*n* = 16) groups. Experimental gingivitis increased in both groups, with rates of 60.0% and 50.0% for FAG and SF, respectively. After the 14‐day intervention, FMPS and FMBS were reduced in both groups compared to the post‐induction phase, confirming the noninferiority of FAG. Specifically, FAG's FMPS was 39.7% ± 13.8% with FMBS at 28.9% ± 16.9%, while SF's FMPS was 43.2% ± 14.9% with FMBS at 21.4% ± 11.9%. No significant FMPS/FMBS differences were observed overtime, and there were also no significant differences in gingivitis rates throughout the trial. Crude and adjusted models, accounting for baseline FMPS, age and gender, reiterated the lack of significant association between outcomes and treatments. The adjusted odds ratio was 0.71 (*p* = 0.662).

**Conclusion:**

This study establishes the noninferiority of fatty acids–based relative to SF mouthrinse in an experimental gingivitis model. Fatty acids offer promising avenues for oral care enhancement, necessitating further investigation and validation in broader real‐life scenarios.

## Clinical Relevance

Scientific Rationale for Study: Despite limited evidence, this RCT explores the efficacy of fatty acids–based mouthrinse in plaque control, addressing a crucial gap in oral care.

Principal Findings: The study shows noninferiority of fatty acids–based mouthrinse compared to stannous fluoride in controlling plaque during experimental gingivitis.

Practical Implications: Clinicians can consider incorporating fatty acids–based mouthrinses as an alternative for plaque control, especially for patients sensitive to fluoride. Further research is warranted to validate these findings and explore long‐term effects.

## 1 Introduction

Oral hygiene plays a crucial role in maintaining oral health and preventing dental diseases, including dental caries and periodontal conditions (Jain et al. 2024). In recent times, there has been a surge in interest surrounding personal oral hygiene, driven by its far‐reaching implications for systemic health (Del Pinto et al. 2021, 2022). Various oral care products have been developed to target plaque accumulation, a primary factor contributing to these oral health issues. Among these products, mouthrinses have gained prominence as convenient adjuncts to daily oral hygiene routines. Traditional mouthrinses often contain antiseptic agents like fluoride, chlorhexidine or essential oils, which have demonstrated significant antimicrobial properties against oral bacteria (Figuero et al. 2020). However, emerging research has explored alternative antimicrobial agents derived from natural sources.

Fatty acids, derived from plant oils or animal fats, have garnered attention due to their potential antimicrobial properties and biocompatibility (Casillas‐Vargas et al. 2021). These compounds exhibit a diverse range of bioactive effects, including antibacterial, anti‐inflammatory, and wound‐healing activities (Casillas‐Vargas et al. 2021). Recent investigations have suggested that certain fatty acids may possess intrinsic antiplaque capabilities, making them promising candidates for oral care formulations (Stańdo and Lewkowicz 2019). By directing their action towards plaque, a sophisticated biofilm primarily composed of bacteria, these fatty acids have the potential to offer an efficacious and comprehensive method for safeguarding oral health, particularly in individuals who have undergone chemotherapy or radiotherapy (Hashemipour et al. 2017). This is attributed to their unique ability to create a protective film on the oral mucosa, thereby contributing to enhanced oral well‐being in this specific cohort (Lessa et al. 2021).

To address the potential benefits of fatty acids in oral care, this study presents a noninferiority randomized clinical trial aimed at evaluating the antiplaque efficacy of a novel fatty acids–based mouthrinse. The primary objective is to determine whether this fatty acids–based formulation (FAG) is noninferior to a conventional stannous fluoride (SF) mouthrinse in reducing plaque accumulation over a defined study period.

SF was chosen as the comparator in this study due to its well‐established reputation as an antiplaque agent (Willumsen et al. 2007; He et al. 2022). It is worth noting that, similar to FAG, SF is not classified as an antibacterial agent (PubChem n.d.‐b; C. B. Huang et al. 2011), distinguishing it from compounds such as chlorhexidine (PubChem n.d.‐a).

Through a human model of induced gingivitis, this investigation seeks to contribute to the expanding body of knowledge regarding natural antimicrobial agents and their potential applications in modern dentistry.

The present study underscores the importance of exploring innovative strategies to enhance oral health, particularly those rooted in naturally derived compounds. By elucidating the antiplaque capacity of FAG mouthrinse, this study aims to provide valuable insights into the development of effective and sustainable oral care interventions, ultimately benefiting individuals striving to achieve optimal oral hygiene and well‐being.

## 2 Methods

### 2.1 Trial Design and Oversight

A monocentric noninferiority randomized controlled clinical trial was structured with the objective of scrutinizing the impact of fatty acids group (FAG, Exposure) vs. SF (Comparator) mouthrinse on full‐mouth plaque score (FMPS) (Outcome) in a cohort with experimental gingivitis (Population). The protocol was approved by the internal review board (Prot. #77593/2021) and registered on ClinicalTrials.gov (NCT04977778).

The present study was conducted in accordance with the Helsinki Declaration as revised in 2013, and the CONSORT checklist was used to report trial information (Supporting Information).

### 2.2 Study Outcomes

The primary outcome of the study is the full‐mouth plaque score (FMPS). Secondary outcomes were full‐mouth bleeding score (FMBS) and the gingivitis rate (defined as bleeding on probing, BoP, > 30%). The noninferiority of the FAG was calculated based on FMPS only.

### 2.3 Sample Size Calculation

Sample size calculation for noninferiority was performed based on established literature (Blackwelder 1982). Computations indicate that if a genuine absence of difference exists between the SF and FAG groups in terms of FMPS (for both at 25% prevalence), a total of 30 patients are necessary to achieve a confidence level of 80%. This ensures that the upper limit of a one‐sided 95% confidence interval, which is equivalently a 90% two‐sided confidence interval, will effectively rule out a difference favoring the SF group exceeding 40% (sealedenvelope.com/power/binary‐noninferior/).

This decision was made without knowledge of the FAG effect on the outcomes.

### 2.4 Trial Population Pre‐Screening

Inclusion criteria were age >=18 years and signed informed consent to participation. Individuals who had received professional oral hygiene within the past 4 months, as well as those who had utilized medications in the past 2 weeks, those with any orthodontic or prosthetic appliances, whether fixed or removable and who had less than 20 natural teeth were excluded. Current smoking, pregnancy, and hormone therapy, and active systemic diseases/conditions were excluding factors. Based on these eligibility criteria, a cohort of 36 healthy, nonsmoking volunteers, spanning an age range of 21–27 years, underwent a pre‐enrollment screening process.

The participants in the study were recruited through a campus campaign. Specifically, this campaign targeted students, faculty, and staff across various departments and faculties within the campus community. The recruitment process was conducted through announcements to ensure a diverse and representative sample from the campus population (E.O.). Individuals were instructed to report any occurrence of side effects.

### 2.5 Trial Procedures

Patients meeting the inclusion criteria were enrolled to undergo subsequent in‐trial procedures.

At baseline (T0), clinical and demographic characteristics were recorded in a cloud‐based system, ensuring anonymity through unique codes. As previously reported, experimental gingivitis was intentionally induced by a 2‐week cessation of teeth brushing (“Dynamics of Red Fluorescent Dental Plaque during Experimental gingivitis—A Cohort Study 2016; Eberhard et al. 2013; Slawik et al. 2011). Specifically, participants were instructed to cease teeth brushing in the right quadrant (Q1 and Q4) for 14 days. Mouthrinses, flossing and wood sticks were forbidden.

**Figure 1:**
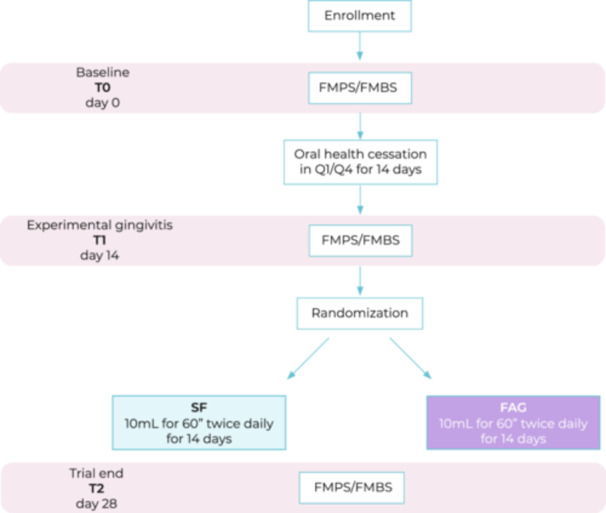
Workflow for the RCT.

Then, clinical features were recorded (T1) and participants were randomly allocated to SF arm (Meridol, Colgate Palmolive S.p.a, Rome IT) or FAG arm (OraLife, Again Life Italia s.r.l., Schio, IT) and received the relative mouthrinse in a standard nonrecognizable packaging. Details of formulations are reported in Table S1. The choice of SF as the control agent, instead of chlorhexidine – widely regarded as the gold standard in the plaque control – was driven by the intent to evaluate the effectiveness of a naturally derived product lacking of antibacterial properties, in comparison with a formulation presenting similar pharmacological characteristics. This approach ensured a more coherent and ethically acceptable comparison within the context of a short‐term trial in healthy participants. Individuals were instructed to use the assigned treatment, consisting of 10 mL mouthrinse use for a duration of 60 s twice daily over the course of 14 days, during which brushing in quadrants Q1 and Q4 was forbidden. Afterwards, clinical indices were recorded (T2) and all the participants underwent full mouth professional oral hygiene and fluoroprophylaxis and resumed the regular daily oral care. All individuals were provided with toothpaste, toothbrushes, and fluoride mouthrinse for home dental hygiene to resume previous oral habits. At the end of the intervention period, participants returned the unused mouthrinse to allow the assessment of actual usage of the product. Trial was completed through September 1, 2021, and July 7, 2023. All procedures are recapitulated in Figure 1.

### 2.6 Randomization Procedures

Eligible patients were randomized in a 1:1 ratio using a block randomization approach (sealedenvelope.com, block size: 4, 6, 8; list length: 40). Allocation concealment was maintained through sealed opaque envelopes containing information about the allocation (SF or FAG). After a patient had given his/her consent to participate in the trial, an envelope was unsealed, revealing the assigned treatment regimen. To ensure blinding for participants and the clinical experimenter (A.B.), an external investigator (E.O.), who was not involved in the clinical and statistical procedures, performed the unsealing and delivered treatments.

### 2.7 Assessment of Clinical Indexes

The same experienced dental hygienist (A.B.) performed all the clinical evaluations. The enrolled individuals underwent charting to record their periodontal conditions. Plaque and BoP were recorded dichotomously (presence/absence) four sites per tooth to calculate the FMPS and the FMBS, respectively (Ainamo and Bay 1975; O'Leary et al. 1972). Specifically, FMPS was assessed following the protocol proposed by Timothy J. O'Leary and colleagues in their seminal article of 1972 (O'Leary et al. 1972). Briefly, a disclosing solution (PlaqSearch – TePe, Malmö, Sweden) was utilized according to the manufacturer's instructions to visualize biofilm accumulation. Following rinsing by the patient, the operator (AB) examined each tooth surface (mesial, distal, facial, lingual) using the probe tip to detect biofilm accumulations at the dentogingival junction, recording findings electronically. Soft accumulations not at the dentogingival junction were not documented, and no differentiation was made between varying degrees of plaque. Subsequently, the FMPS was calculated by dividing the number of plaque‐containing surfaces by the total available surfaces.

All sites were monitored with a UNC‐15 periodontal probe at a force of approximately 0.25 N. FMPS and FMBS were calculated as the percentage of sites with detectable BoP or plaque on the dentition. Presence of plaque‐induced gingivitis was defined as BoP > 30% (Trombelli et al. 2018).

### 2.8 Statistical Analysis

Before conducting the statistical analysis, a data validation process was undertaken involving double data entry and proofreading to ensure accuracy. Continuous variables were succinctly presented as mean and standard deviation (SD), while categorical variables were concisely represented as proportions within tables. In data visualization, the mean and standard error (SE) were incorporated.

To scrutinize continuous data, both parametric and nonparametric methods, employing the *t*‐test and Wilcoxon rank sum test, respectively, were adopted. The assessment of the odds ratio (OR) for gingivitis was accomplished through logistic regression, presenting the results with their corresponding 95% confidence intervals (CIs). A crude and an adjusted model (age, sex, and baseline FMPS) were generated. Statistical significance was attributed to data with P values less than 0.05, signifying the threshold for noteworthy findings. The entirety of statistical analyses and data visualization were generated using R software, version 4.2.1. The complete R code is provided at github. com/PietropaoliLab/FAG.

## 3 Results

**Figure 2:**
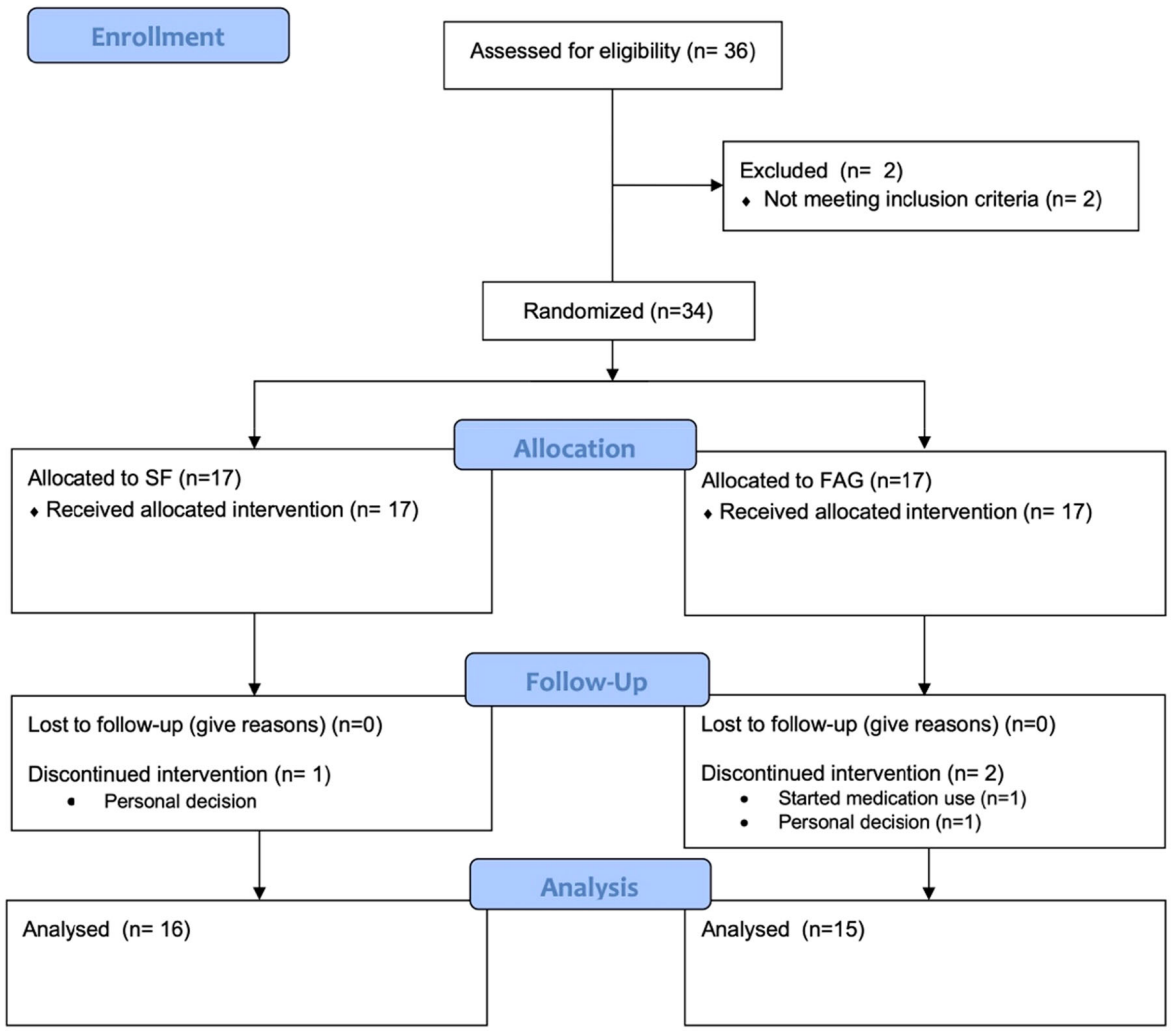
CONSORT flow diagram outlines the progress of participants through the phases of a randomized clinical trial comparing two mouthrinses (SF and FAG) for the treatment of gingivitis.

After a thorough pre‐screening phase, a total of 34 Caucasian individuals (100%) were successfully enrolled in the study (Figure 2). Three participants discontinued the intervention due to personal reasons and were subsequently excluded from the analysis. Thirty‐one individuals successfully completed the full protocol, resulting in a dropout rate of 8.8%. Of the enrolled participants, fifteen were assigned to the FAG arm, while sixteen were allocated to the SF arm. Comprehensive clinical and demographic information is presented in Table 1, while Table S2 provides the same data analyzed using nonparametric tests. All the expected amount of mouthrinse was used. No side effects were reported by study participants.

**Table 1:** Clinical demographic characteristics. *p* values reported results of parametric test (i.e., *t*‐test or chi‐squared test).

		Overall	FAG	SF	*p* value
	*n*	31	15	16	
	Male (%)	13 (41.9)	6 (40.0)	7 (43.8)	
	Age (mean (SD))	22.90 (1.58)	22.93 (1.87)	22.88 (1.31)	0.920
T0	FMBS (mean (SD))	19.08 (12.22)	21.87 (10.90)	16.46 (13.14)	0.224
	FMPS (mean (SD))	31.29 (21.17)	34.20 (16.07)	28.56 (25.27)	0.468
	Gingivitis (%)	4 (12.9)	1 (6.7)	3 (18.8)	0.641
T1	FMBS (mean (SD))	32.47 (14.15)	36.92 (13.33)	28.29 (14.00)	0.09
	FMPS (mean (SD))	50.48 (13.39)	52.00 (11.49)	49.06 (15.19)	0.551
	Gingivitis (%)	17 (54.8)	9 (60.0)	8 (50.0)	0.843
T2	FMBS (mean (SD))	25.02 (14.78)	28.86 (16.90)	21.43 (11.92)	0.166
	FMPS (mean (SD))	41.48 (14.26)	39.67 (13.77)	43.19 (14.94)	0.501
	Gingivitis (%)	11 (35.5)	6 (40.0)	5 (31.2)	0.894

The mean age of the participants was 22.9 ± 1.58 years, with a majority being female (57.1%). It is worth noting that the baseline gingivitis rate was not different between the two arms. Specifically, the baseline gingivitis rate within the FAG group was 6.67% (95% CI 0.34–34.0), while the SF group exhibited a baseline rate of 18.75% (95% CI: 5.0–46.3). The calculated between‐groups difference was 12.08% (95% CI: 4.1–17.3; *p*‐value: 0.641).

**Figure 3:**
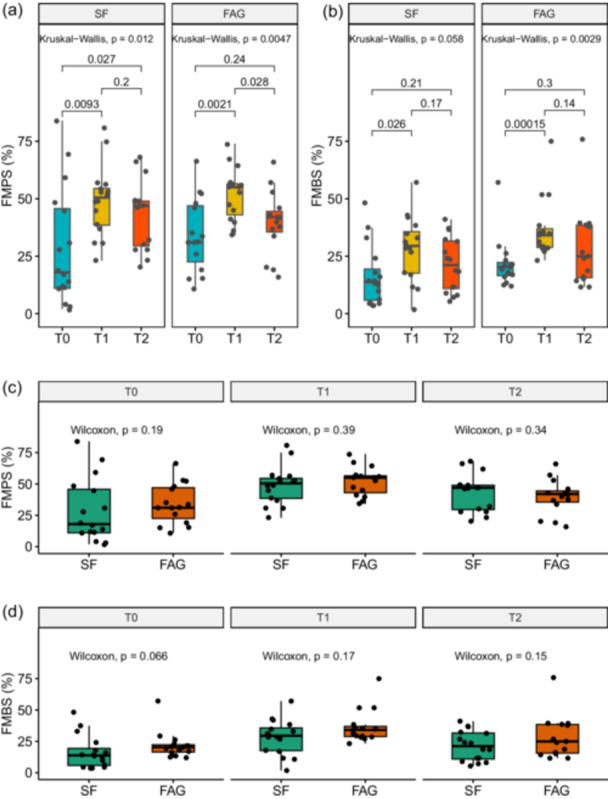
Comparative analyses within and between groups. Within‐treatment analyses of FMPS and FMPS are reported in (a) and (b), respectively. Between‐treatment analyses are reported in (c) and (d). Mean and standard errors are reported. *p* values correspond to nonparametric tests. Bonferroni correction for multiple comparisons was applied as appropriate.

Upon the conclusion of the 14‐day period of abstaining from oral hygiene practices, the gingivitis rate significantly increased to 60.0% (95% CI: 32.9–82.5) in the FAG group and to 50.0% (95% CI: 28.0–72.0) in the SF group (*p* = 0.843). Correspondingly, both FMPS and FMBS significantly increased in both groups, as depicted in Figure 3.

Within‐groups comparisons showed that, at the end of the 14‐day mouthrinse regimen, FMPS was lower than in T1 in the group allocated to FAG (Figure 3a, Wilcoxon *p*‐value = 0.028), while a nonsignificant reduction in FMBS when comparing T2 vs. T1 was found in both groups (Figure 3b). However, between‐group comparisons from T0 to T2 did not reveal any substantial differences in bleeding or plaque burden over time (Figure 3c, d).

At the end of the trial, the observed gingivitis rate was 40.0% (95% CI: 17.5–67.1) within the FAG group and 31.25% (95% CI: 12.1–58.5) within the SF group. The between‐groups difference was quantified at 8.75% (95% CI: −31.3 to 48.8; *p*‐value: 0.894).

Subsequent analysis, encompassing both crude and adjusted logistic models, failed to demonstrate statistical significance between the gingivitis rate and the treatment administered. The crude OR was computed at 0.68 (95% CI: 0.15–3.00; *p* value = 0.612), and after adjusting for baseline FMPS, age and gender, the adjusted OR was found to be 0.71 (95% CI: 0.14–3.33; *p* value = 0.662).

## 4 Discussion

In the context of this trial, we have established the noninferiority of FAG in comparison to SF with respect to reductions in FMPS and FMBS, as well as in the context of lowering the gingivitis rate within the experimental framework. These findings harmoniously align with existing literature, which indicates that fatty acid mouthrinses can serve as effective alternatives for managing plaque‐dependent gingivitis (Casillas‐Vargas et al. 2021).

These formulations may represent a promising avenue for future studies involving individuals undergoing cancer therapy, especially in light of previous findings showing good tolerability of fatty acid–based products. Notably, these patients reported significantly lower levels of oral cavity and throat discomfort and irritation during therapies, thus contributing to an improved quality of life and performance in essential activities such as eating, sleeping, and swallowing liquids (Hashemipour et al. 2017; Lessa et al. 2021). The salient mechanisms driving these positive outcomes may be attributed to fatty acids' potential to expedite wound healing (McDaniel et al. 2008). Additionally, the antibacterial and anti‐inflammatory properties inherent to the fatty acids family (Yang et al. 2017; W.‐C. Huang et al. 2014), coupled with recent insights into inhibition of DNA/RNA replication, protein synthesis, cytoplasmic membrane disruption, and metabolic route inhibition, further illuminate the multifaceted role of fatty acids within the dental plaque biofilm (Casillas‐Vargas et al. 2021). However, the short duration of FAG mouthrinse use in the current study precludes drawing conclusions about its long‐term safety or efficacy in oncologic patients. Hence, these preliminary findings should be interpreted cautiously and explored further in dedicated studies.

From an oral perspective, omega‐3 fatty acids have demonstrated efficacy in ameliorating clinical attachment loss and probing depth during nonsurgical therapy for individuals afflicted by periodontitis (Maybodi et al. 2022). Parallel to our own findings, a parallel randomized clinical trial reported no significant difference in terms of FMBS reduction between fatty acid and standard control groups containing soybean oil, used as an adjunct therapy during professional dental hygiene (Maybodi et al. 2022). However, other investigations have highlighted a notable reduction in BoP within the fatty acid group compared to the control group (Stańdo et al. 2020). Notwithstanding that evidence, the use of adjunctives needs to be evaluated case by case in light of EFP S3 guidelines (Sanz et al. 2020; Herrera et al. 2022). While adjunctive rinsing solutions provide some support, they are not substitutes for thorough mechanical biofilm removal, which remains crucial for controlling gingivitis and periodontitis. A personalized approach of mechanical and adjunctive methods is key to maintaining oral health. (Sanz et al. 2020; Herrera et al. 2022).

While this study unveils crucial insights, it is prudent to acknowledge its limitations when interpreting the results. The limited sample size and the relatively homogenous cohort, comprising predominantly young Caucasian individuals without periodontitis, could affect the generalizability of the findings. Additionally, the volunteer‐based recruitment approach may introduce selection bias, and the relatively short treatment duration and follow‐up period warrant careful consideration. Furthermore, pertinent data on participants' oral habits before trial initiation were not collected. To ensure a rigorously standardized and controlled experimental setting, mechanical tooth brushing was suspended. Eliminating mechanical action allowed for the minimization of confounding factors related to individual oral hygiene practices and enabled a more accurate attribution of observed effects to the tested intervention. While the inclusion of additional groups—such as one undergoing mechanical cleaning alone or a combination protocol—could have provided further comparative insights, such analyses fell outside the scope of this noninferiority trial, which was specifically designed to evaluate chemical control in isolation. These aspects warrant further investigation in future studies. Nevertheless, this study boasts several strengths. Additionally, when interpreting the results, it is important to consider the relatively short treatment duration (14 days) and the absence of patient‐centered outcomes such as taste, feeling of freshness, and burning sensation. It should be noted, however, that previous trials used the same in‐trial time frame and documented early formation of plaque after the abolishment of mechanical tooth cleaning (Parkinson et al. 2018; Furuichi et al. 1992). A meticulous evaluation ensured the absence of confounding factors such as diseases, medication history, prior professional dental hygiene, and pregnancy. Furthermore, a rigorous randomization process balanced groups in terms of gender, age, baseline FMPS, FMBS, and gingivitis rate. The clinical evaluations were performed by a single operator, and both the operator and participants remained blinded to the treatment allocation. Robust statistical analyses were underpinned by both parametric and nonparametric approaches, with crude and adjusted logistic regression models validating group differences, excluding potential age and gender biases (Pietropaoli et al. 2023).

## 5 Conclusion

In conclusion, this study has demonstrated the noninferiority of FAG compared to SF. Our findings support the use of FAG in plaque control measures, highlighting its potential as a viable alternative to SF. These results emphasize the importance of exploring alternative agents in oral health care and pave the way for further investigations into the efficacy and safety of FAG in broader contexts. Moving forward, additional studies are warranted to validate and extend these outcomes, ultimately enhancing our understanding of effective antiplaque strategies.

## Author Contributions

Serena Altamura and Eleonora Ortu made equal contributions to this study. Davide Pietropaoli conceptualized and designed the study, conducted statistical analyses, authored the manuscript, performed data visualization, and interpreted the results. Antonella Barone, Eleonora Ortu, and Serena Altamura engaged in the trial phases and initial manuscript proofreading. Antonella Barone, Annalisa Monaco, Martina Cardisciani, and Sara Di Nicolantonio revised the manuscript. All authors have accepted responsibility for the entirety of the work. Additionally, all authors have reviewed and endorsed the final version of the manuscript.

## Conflicts of Interest

D.P. received honoraria from Colgate Palmolive, which is acknowledged here for transparency purposes. All the other authors declare no conflicts of interest.

## Supporting information

cre2.20250265‐File005.

cre2.20250265‐File006.

cre2.20250265‐File007.docx.

cre2.20250265‐File008.

## Data Availability

The data supporting this study's findings are available from the corresponding author upon reasonable request.
