# Supplementary material for: Noninferiority Randomized Controlled Clinical Trial Assessing the Antiplaque Efficacy of Fatty Acids–Based Mouthrinse
Source: Clin Exp Dent Res. 2025 Jul 9;11(4):e70171. doi: 10.1002/cre2.70171 (PMC12239514; doi:10.1002/cre2.70171)
Supplement: Supplementary file 2 — cre2.20250265‐File006. [file CRE2-11-e70171-s003.pdf]

Non-Inferiority Randomized Controlled Clinical Trial Assessing the Antiplaque Efficacy of Fatty Acids-Based Mouthrinse

R Markdown

Antonella Barone*, Eleonora Ortu*, Mario Giannoni, Annalisa Monaco, Serena Altamura and Davide pietropaoli

These Authors contributed equally to the work

Corresponding author: Davide Pietropaoli DDS, PhD

University of L'Aquila R.L. Montalcini Building, (Delta 6),

via Giuseppe Petrini, Coppito,L'Aquila, 67100.

E-mail address: davide.pietropaoli@univaq.it

Load libraries and dataset

```
library(here)
```

```
## here() starts at /Users/davidepietropaoli/Il mio Drive/01- UNIVERSITA
```

```
library(ggpubr)
```

```
## Loading required package: ggplot2
```

```
library(tableone)
library(readxl)
library(reshape2)
library(stringr)
library(patchwork)


#Import data
Mouthwash <- read_excel(here("AQ Projects/AgainLife/Data/Mouthwash.xlsx"))
```

Generating Table 1

```
# Table One
## Vector of variables to summarize
myVars <- c("Group", "Gender", "Age", "FMBS_T0", "FMPS_T0", "BOP30_T0", "FMBS_T1",
            "FMPS_T1", "BOP30_T1", "FMBS_T2", "FMPS_T2", "BOP_30_T2")

## Create a TableOne object
TableOne <- CreateTableOne(vars = myVars, strata = "Group",  addOverall = TRUE, data = Mouthwash)
```

```
kableone(TableOne, quote = FALSE, noSpace = TRUE, showAllLevels = TRUE)
```

	level	Overall	FAG	FLUORO	p	test
n		31	15	16		
Group (%)	FAG	15 (48.4)	15 (100.0)	0 (0.0)	<0.001	
	FLUORO	16 (51.6)	0 (0.0)	16 (100.0)		
Gender (%)	F	18 (58.1)	9 (60.0)	9 (56.2)	1.000	
	M	13 (41.9)	6 (40.0)	7 (43.8)		
Age (mean (SD))		22.90 (1.58)	22.93 (1.87)	22.88 (1.31)	0.920	
FMBS_T0 (mean (SD))		19.08 (12.22)	21.87 (10.90)	16.46 (13.14)	0.224	
FMPS_T0 (mean (SD))		31.29 (21.17)	34.20 (16.07)	28.56 (25.27)	0.468	
BOP30_T0 (%)	Gingivitis	4 (12.9)	1 (6.7)	3 (18.8)	0.641	
	No gingivitis	27 (87.1)	14 (93.3)	13 (81.2)		
FMBS_T1 (mean (SD))		32.47 (14.15)	36.92 (13.33)	28.29 (14.00)	0.090	
FMPS_T1 (mean (SD))		50.48 (13.39)	52.00 (11.49)	49.06 (15.19)	0.551	
BOP30_T1 (%)	Gingivitis	17 (54.8)	9 (60.0)	8 (50.0)	0.843	
	No gingivitis	14 (45.2)	6 (40.0)	8 (50.0)		
FMBS_T2 (mean (SD))		25.02 (14.78)	28.86 (16.90)	21.43 (11.92)	0.166	
FMPS_T2 (mean (SD))		41.48 (14.26)	39.67 (13.77)	43.19 (14.94)	0.501	
BOP_30_T2 (%)	Gingivitis	11 (35.5)	6 (40.0)	5 (31.2)	0.894	
	No gingivitis	20 (64.5)	9 (60.0)	11 (68.8)		

Table 1 with non-parametric testing

Specify summary statistics to use medians and IQRs

```
kableone(TableOne,
         showAll = TRUE,  # Show all variables
         quote = FALSE,   # Do not quote factor levels
         noSpaces = TRUE, # No extra spaces in the output
         smd = TRUE,      # Show standardized mean differences
         test = TRUE,     # Perform statistical tests
         pDigits = 3,     # Digits for p-values
         nonnormal = TRUE, # Specify non-normal distributions
         nudge = TRUE      # Adjust formatting of the table
)
```

	level	Overall	FAG	FLUORO	p	test	SMD
n		31	15	16			
Group (%)	FAG	15 (48.4)	15 (100.0)	0 (0.0)	<0.001		NaN
	FLUORO	16 (51.6)	0 (0.0)	16 (100.0)			
Gender (%)	F	18 (58.1)	9 (60.0)	9 (56.2)	1.000		0.076
	M	13 (41.9)	6 (40.0)	7 (43.8)			
Age (median [IQR])		23.00 [21.50, 24.00]	23.00 [21.00, 24.50]	23.00 [22.00, 24.00]	0.888	nonnorm	0.036
FMBS_T0 (median [IQR])		16.96 [12.50, 22.32]	20.36 [16.52, 22.32]	13.84 [5.80, 19.42]	0.063	nonnorm	0.448
FMPS_T0 (median [IQR])		31.00 [14.50, 47.00]	31.00 [22.50, 47.00]	18.00 [11.00, 45.75]	0.185	nonnorm	0.266
BOP30_T0 (%)	Gingivitis	4 (12.9)	1 (6.7)	3 (18.8)	0.641		0.369
	No gingivitis	27 (87.1)	14 (93.3)	13 (81.2)			
FMBS_T1 (median [IQR])		31.25 [27.64, 36.97]	34.11 [28.88, 36.97]	29.46 [17.63, 35.71]	0.160	nonnorm	0.632
FMPS_T1 (median [IQR])		53.00 [40.50, 56.00]	55.00 [43.00, 56.50]	50.50 [38.50, 54.50]	0.384	nonnorm	0.218
BOP30_T1 (%)	Gingivitis	17 (54.8)	9 (60.0)	8 (50.0)	0.843		0.202
	No gingivitis	14 (45.2)	6 (40.0)	8 (50.0)			
FMBS_T2 (median [IQR])		24.11 [12.95, 36.88]	25.00 [15.64, 38.48]	21.16 [10.94, 31.65]	0.149	nonnorm	0.508
FMPS_T2 (median [IQR])		42.00 [31.00, 49.00]	42.00 [35.50, 44.50]	47.00 [29.50, 49.00]	0.332	nonnorm	0.245
BOP_30_T2 (%)	Gingivitis	11 (35.5)	6 (40.0)	5 (31.2)	0.894		0.183
	No gingivitis	20 (64.5)	9 (60.0)	11 (68.8)			

Data visualization

Preparing data

```
m <- melt(Mouthwash[c(4:6, 8,9, 11, 12)], id.vars = c("Group"))
m$Timing <- str_sub(m$variable, start = -2)

m$variable <- gsub("_T0", "", m$variable)
m$variable <- gsub("_T1", "", m$variable)
m$variable <- gsub("_T2", "", m$variable)

my_comparisons <- list( c("T0", "T1"), c("T1", "T2"), c("T0", "T2") )
m$Group <- factor(m$Group, levels = c("FLUORO", "FAG"), labels = c("SF", "FAG"))
```

FMPS

```
p <- ggboxplot(subset(m, variable == "FMPS"), x = "Timing", y = "value",
          fill = "Timing", palette =c("#00AFBB", "#E7B800", "#FC4E07"),
          color = "gray30",
          width = .4,
          facet.by = c("Group"),
          add = "jitter")+
  labs(title = "A", x= NULL, y = "FMPS (%)")+
  scale_y_continuous(breaks = c(0, 25, 50, 75))+
  coord_cartesian(ylim = c(0, 120))+
  theme(legend.position = "none")+
stat_compare_means(comparisons = my_comparisons, size = 3.5)+ # Add pairwise comparisons p-value
stat_compare_means(label.y = 118, size = 3)                   # Add global p-value
```

FMBS

```
p1 <- ggboxplot(subset(m, variable == "FMBS"), x = "Timing", y = "value",
               fill = "Timing", palette =c("#00AFBB", "#E7B800", "#FC4E07"),
               color = "gray30",
               width = .4,
               facet.by = c("Group"),
               add = "jitter")+
  labs(title = "B", x= NULL, y = "FMBS (%)")+
  scale_y_continuous(breaks = c(0, 25, 50, 75))+
  coord_cartesian(ylim = c(0, 120))+
  theme(legend.position = "none")+
stat_compare_means(comparisons = my_comparisons, size = 3.5)+ # Add pairwise comparisons p-value
stat_compare_means(label.y = 118, size = 3)                   # Add global p-value
```

Between group (SF vs FAG) according time

FMPS

```
p2 <- ggboxplot(data = subset(m, variable == "FMPS"),
          x = 'Group', y = 'value',
          fill = 'Group', palette = 'Dark2',
          width = .5,
          add = "jitter") +
  facet_wrap(.~Timing) +
  labs(title = "C", x= NULL, y = "FMPS (%)")+
  coord_cartesian(ylim = c(0, 110))+
  scale_y_continuous(breaks = c(0, 25, 50, 75))+
  stat_compare_means(label.y = 90, size = 3.5) +
  theme(legend.position = "none")
```

FMPS

```
p3 <- ggboxplot(data = subset(m, variable == "FMBS"),
                x = 'Group', y = 'value',
                fill = 'Group', palette = 'Dark2',
                width = .5,
                add = "jitter") +
  facet_wrap(.~Timing) +
  labs(title = "D", x= NULL, y = "FMBS (%)")+
  coord_cartesian(ylim = c(0, 110))+
  scale_y_continuous(breaks = c(0, 25, 50, 75))+
  stat_compare_means(label.y = 90, size = 3.5) +
  theme(legend.position = "none")
```

Combining plots

```
design <- "AABB
           CCCC
           DDDD"
Fig1 <- p + p1 + p2 + p3 + plot_layout(design = design, heights = c(2, 1, 1))
print(Fig1)
```

```
## Warning in wilcox.test.default(c(14, 19, 48, 28, 2, 69, 59, 45, 3, 11, 4, :
## cannot compute exact p-value with ties
```

```
## Warning in wilcox.test.default(c(14, 19, 48, 28, 2, 69, 59, 45, 3, 11, 4, :
## cannot compute exact p-value with ties
```

```
## Warning in wilcox.test.default(c(57, 53, 49, 52, 31, 54, 75, 45, 31, 39, :
## cannot compute exact p-value with ties
```

```
## Warning in wilcox.test.default(c(35, 53, 11, 52, 34, 31, 48, 19, 26, 46, :
## cannot compute exact p-value with ties
```

```
## Warning in wilcox.test.default(c(35, 53, 11, 52, 34, 31, 48, 19, 26, 46, :
## cannot compute exact p-value with ties
```

```
## Warning in wilcox.test.default(c(56, 64, 53, 74, 40, 55, 57, 34, 41, 55, :
## cannot compute exact p-value with ties
```

```
## Warning in wilcox.test.default(c(9.82, 37.5, 48.21, 4.46, 4.46, 12.5, 17.86, :
## cannot compute exact p-value with ties
```

```
## Warning in wilcox.test.default(c(9.82, 37.5, 48.21, 4.46, 4.46, 12.5, 17.86, :
## cannot compute exact p-value with ties
```

```
## Warning in wilcox.test.default(c(42.86, 26.79, 41.96, 33, 17.86, 16.96, :
## cannot compute exact p-value with ties
```

```
## Warning in wilcox.test.default(c(19.29, 20.36, 12.5, 11.97, 21.43, 57.14, :
## cannot compute exact p-value with ties
```

```
## Warning in wilcox.test.default(c(19.29, 20.36, 12.5, 11.97, 21.43, 57.14, :
## cannot compute exact p-value with ties
```

```
## Warning in wilcox.test.default(c(75, 28.57, 34.82, 23.21, 27.04, 51.79, :
## cannot compute exact p-value with ties
```

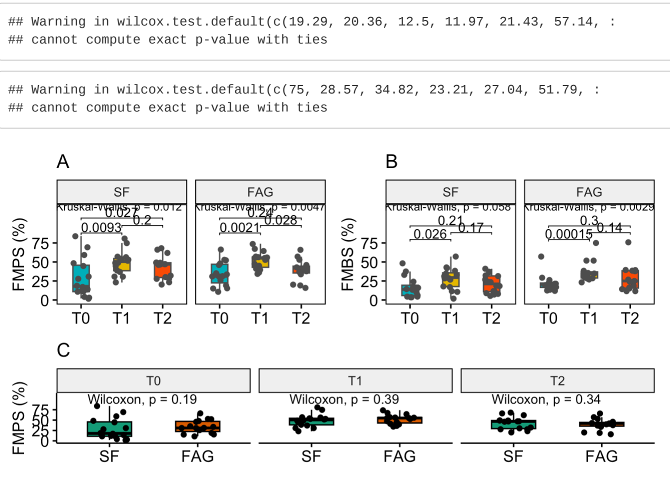

Save Figure 1

```
ggsave(filename = here("AQ Projects/AgainLife/Manuscript/Figures/Figure 1.png"), plot = Fig1,
       device = "png", units = "px", height = 3000, width = 2250, dpi = 300)
```

```
## Warning in wilcox.test.default(c(14, 19, 48, 28, 2, 69, 59, 45, 3, 11, 4, :
## cannot compute exact p-value with ties
```

```
## Warning in wilcox.test.default(c(14, 19, 48, 28, 2, 69, 59, 45, 3, 11, 4, :
## cannot compute exact p-value with ties
```

```
## Warning in wilcox.test.default(c(57, 53, 49, 52, 31, 54, 75, 45, 31, 39, :
## cannot compute exact p-value with ties
```

```
## Warning in wilcox.test.default(c(35, 53, 11, 52, 34, 31, 48, 19, 26, 46, :
## cannot compute exact p-value with ties
```

```
## Warning in wilcox.test.default(c(35, 53, 11, 52, 34, 31, 48, 19, 26, 46, :
## cannot compute exact p-value with ties
```

```
## Warning in wilcox.test.default(c(56, 64, 53, 74, 40, 55, 57, 34, 41, 55, :
## cannot compute exact p-value with ties
```

```
## Warning in wilcox.test.default(c(9.82, 37.5, 48.21, 4.46, 4.46, 12.5, 17.86, :
## cannot compute exact p-value with ties
```

```
## Warning in wilcox.test.default(c(9.82, 37.5, 48.21, 4.46, 4.46, 12.5, 17.86, :
## cannot compute exact p-value with ties
```

```
## Warning in wilcox.test.default(c(42.86, 26.79, 41.96, 33, 17.86, 16.96, :
## cannot compute exact p-value with ties
```

```
## Warning in wilcox.test.default(c(19.29, 20.36, 12.5, 11.97, 21.43, 57.14, :
## cannot compute exact p-value with ties
```

```
## Warning in wilcox.test.default(c(19.29, 20.36, 12.5, 11.97, 21.43, 57.14, :
## cannot compute exact p-value with ties
```

```
## Warning in wilcox.test.default(c(75, 28.57, 34.82, 23.21, 27.04, 51.79, :
## cannot compute exact p-value with ties
```

Compute Odds Ratio and 95% CI

```
# Odds Ratio
Mouthwash$Outcome <- ifelse(Mouthwash$BOP_30_T2 == "Gingivitis", 1, 0)
model <- glm(Outcome ~ Group, data = Mouthwash, family = binomial)
summary(model)
```

```
##
## Call:
## glm(formula = Outcome ~ Group, family = binomial, data = Mouthwash)
##
## Coefficients:
##             Estimate Std. Error z value Pr(>|z|)
## (Intercept)  -0.4055     0.5270  -0.769    0.442
## GroupFLUORO  -0.3830     0.7541  -0.508    0.612
##
## (Dispersion parameter for binomial family taken to be 1)
##
##     Null deviance: 40.324  on 30  degrees of freedom
## Residual deviance: 40.065  on 29  degrees of freedom
## AIC: 44.065
##
## Number of Fisher Scoring iterations: 4
```

```
## odds ratios and 95% CI
exp(cbind(OR = coef(model), confint(model)))
```

```
## Waiting for profiling to be done...
```

```
##                    OR     2.5 %   97.5 %
## (Intercept) 0.6666667 0.2234795 1.848722
## GroupFLUORO 0.6818182 0.1493450 2.997447
```

```
sessionInfo()
```

```
## R version 4.3.2 (2023-10-31)
## Platform: aarch64-apple-darwin20 (64-bit)
## Running under: macOS Sonoma 14.5
##
## Matrix products: default
## BLAS:   /Library/Frameworks/R.framework/Versions/4.3-arm64/Resources/lib/libRblas.0.dylib
## LAPACK: /Library/Frameworks/R.framework/Versions/4.3-arm64/Resources/lib/libRlapack.dylib;  LAPACK version 3.1
1.0
##
## locale:
## [1] en_US.UTF-8/en_US.UTF-8/en_US.UTF-8/C/en_US.UTF-8/en_US.UTF-8
##
## time zone: Europe/Rome
## tzcode source: internal
##
## attached base packages:
## [1] stats     graphics  grDevices utils     datasets  methods   base
##
## other attached packages:
## [1] patchwork_1.2.0 stringr_1.5.1   reshape2_1.4.4  readxl_1.4.3
## [5] tableone_0.13.2 ggpubr_0.6.0    ggplot2_3.5.0   here_1.0.1
##
## loaded via a namespace (and not attached):
##  [1] gtable_0.3.4       xfun_0.42          bslib_0.6.1        rstatix_0.7.2
##  [5] lattice_0.22-5     vctrs_0.6.5        tools_4.3.2        generics_0.1.3
##  [9] tibble_3.2.1       proxy_0.4-27       fansi_1.0.6        highr_0.10
## [13] pkgconfig_2.0.3    Matrix_1.6-5       RColorBrewer_1.1-3 lifecycle_1.0.4
## [17] farver_2.1.1       compiler_4.3.2     textshaping_0.3.7  munsell_0.5.0
## [21] mitools_2.4        carData_3.0-5      survey_4.4-2       htmltools_0.5.7
## [25] class_7.3-22       sass_0.4.8         yaml_2.3.8         pillar_1.9.0
## [29] car_3.1-2          jquerylib_0.1.4    tidyr_1.3.1        MASS_7.3-60.0.1
## [33] cachem_1.0.8       abind_1.4-5        tidyselect_1.2.0   digest_0.6.34
## [37] stringi_1.8.3      dplyr_1.1.4        purrr_1.0.2        forcats_1.0.0
## [41] splines_4.3.2      labelled_2.12.0    rprojroot_2.0.4    fastmap_1.1.1
## [45] grid_4.3.2         colorspace_2.1-0   cli_3.6.2          magrittr_2.0.3
## [49] survival_3.5-8     utf8_1.2.4         broom_1.0.5        e1071_1.7-14
## [53] withr_3.0.0        scales_1.3.0       backports_1.4.1    rmarkdown_2.25
## [57] ggsignif_0.6.4     cellranger_1.1.0   ragg_1.2.7         zoo_1.8-12
## [61] hms_1.1.3          evaluate_0.23      knitr_1.45         haven_2.5.4
## [65] rlang_1.1.3        Rcpp_1.0.12        glue_1.7.0         DBI_1.2.2
## [69] rstudioapi_0.15.0  jsonlite_1.8.8     R6_2.5.1           plyr_1.8.9
## [73] systemfonts_1.0.5
```
