# Supplementary material for: Noninferiority Randomized Controlled Clinical Trial Assessing the Antiplaque Efficacy of Fatty Acids–Based Mouthrinse
Source: Clin Exp Dent Res. 2025 Jul 9;11(4):e70171. doi: 10.1002/cre2.70171 (PMC12239514; doi:10.1002/cre2.70171)
Supplement: Supplementary file 3 — cre2.20250265‐File007.docx. [file CRE2-11-e70171-s004.docx]

**Supplementary Table 1: Details of mouthrinse composition**

| **SF** | **FAG** |
| --- | --- |
| Aqua; xylitol; PVP; PEG-40 hydrogenated castor oil; olaflur; aroma; stannous fluoride; sodium saccharin; C.I. 42051. Contains amine fluoride and sanoc fluoride (250 ppm). | 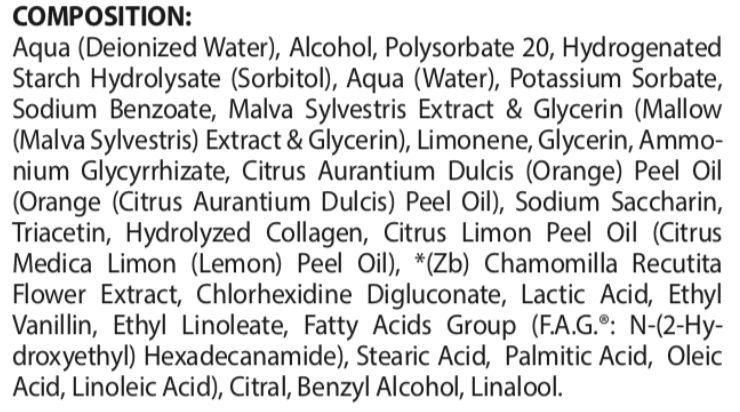 |
