# Supplementary material for: Noninferiority Randomized Controlled Clinical Trial Assessing the Antiplaque Efficacy of Fatty Acids–Based Mouthrinse
Source: Clin Exp Dent Res. 2025 Jul 9;11(4):e70171. doi: 10.1002/cre2.70171 (PMC12239514; doi:10.1002/cre2.70171)
Supplement: Supplementary file 4 — cre2.20250265‐File008. [file CRE2-11-e70171-s002.docx]

**S Table 2. Clinical demographic characteristics.** P values reported results of non-parametric test (i.e. Wilcoxon rank-sum test or Fisher's exact test)

|  |  | **Overall** | **FAG** | **SF** | **P value** |
| --- | --- | --- | --- | --- | --- |
|  | n | 31 | 15 | 16 |  |
|  | Female (%) | 18 (58.1) | 9 (60.0) | 9 (56.2) | 1.000 |
|  | Male (%) | 13 (41.9) | 6 (40.0) | 7 (43.8) |  |
|  | Age (median [IQR]) | 23.00 [21.50, 24.00] | 23.00 [21.00, 24.50] | 23.00 [22.00, 24.00] | 0.888 |
| T0 | FMBS (median [IQR]) | 16.96 [12.50, 22.32] | 20.36 [16.52, 22.32] | 13.84 [5.80, 19.42] | 0.063 |
|  | FMPS (median [IQR]) | 31.00 [14.50, 47.00] | 31.00 [22.50, 47.00] | 18.00 [11.00, 45.75] | 0.185 |
|  | Gingivitis (%) | 4 (12.9) | 1 (6.7) | 3 (18.8) | 0.641 |
| T1 | FMBS (median [IQR]) | 31.25 [27.64, 36.97] | 34.11 [28.88, 36.97] | 29.46 [17.63, 35.71] | 0.160 |
|  | FMPS (median [IQR]) | 53.00 [40.50, 56.00] | 55.00 [43.00, 56.50] | 50.50 [38.50, 54.50] | 0.384 |
|  | Gingivitis (%) | 17 (54.8) | 9 (60.0) | 8 (50.0) | 0.843 |
| T2 | FMBS (median [IQR]) | 24.11 [12.95, 36.88] | 25.00 [15.64, 38.48] | 21.16 [10.94, 31.65] | 0.149 |
|  | FMPS (median [IQR]) | 42.00 [31.00, 49.00] | 42.00 [35.50, 44.50] | 47.00 [29.50, 49.00] | 0.332 |
|  | Gingivitis (%) | 11 (35.5) | 6 (40.0) | 5 (31.2) | 0.894 |
